# Acknowledgment to the Reviewers of *Geriatrics* in 2022

**DOI:** 10.3390/geriatrics8010016

**Published:** 2023-01-18

**Authors:** Geriatrics Editorial Office

**Affiliations:** MDPI AG, St. Alban-Anlage 66, 4052 Basel, Switzerland

High-quality academic publishing is built on rigorous peer review. *Geriatrics* was able to uphold its high standards for published papers due to the outstanding efforts of our reviewers. Thanks to the efforts of our reviewers in 2022, the median time to first decision was 16 days and the median time to publication was 39.5 days. Regardless of whether the articles they examined were ultimately published, the editors would like to express their appreciation and thank the following reviewers for the time and dedication that they have shown *Geriatrics*:
Abdelkader, HamdyLuna-Perejón, FranciscoAbedpoor, NavidMa, NingAcanfora, DomenicoMacDonell, SueAchenbaum, AndrewMacoir, JoelAlexa, Ioana DanaMadinabeitia, IkerAmirabdollahian, FarzadMarano, PietroAndersson, Jonas E.Marchesini, MaurizioAndo, TakafumiMarina, MichelAndrew, Melissa K.Marincu, IosifAnton, NicoletaMarques, DiogoArias, Ana VictoriaMarques, Isabela AlvesAucella, FilippoMarston, Hannah R.Aung, Wai PhyoMartin, PeterAzzolino, DomenicoMartínez-Arnau, Francisco MiguelBaceviciene, MigleMartínez-Ramón, Juan PedroBaixinho, Cristina LavaredaMartín-García, Antonio VíctorBasishvili, TamarMartins, Anabela CorreiaBasu, Jayasree (Joy)Matveeva, MariaBaumrucker, StevenMazaheri, RezaBerkowsky, Ronald W.Mazza, LilianaBernstein, MelissaMazzeo, SalvatoreBilajac, LovorkaMcDonnell-Naughton, MaryBirková, AnnaMechili, Enkeleint A.Birt, LindaMendoza-Nuñez, Victor ManuelBonilla, PatriciaMerellano-Navarro, EugenioBori, GuillemMichel, Jean-PierreBoshnjaku, ArbenMitchell, Aaron P.Bozic, JoskoMoermans, VincentBrogna, BarbaraMohan, DeviBurgio, FrancescaMomtaz, Yadollah AbolfathiByeon, HaewonMondal, JayantaCaballero-Mora, Maria AngelesMone, PasqualeCaffò, Alessandro OronzoMonsuez, Jean-JacquesCarbonelli, CristianoMorales-Gázquez, María J.Cardona, FernandoMoreira, Rafaella PessoaCasini, GiambertoMoreira, TeresaCastel, AlanMorozova, Margarita A.Catic, Angela GeorgiaMucientes, ArkaitzCerdá, BegoñaMulligan, Angela A.Cerejeira, JoaquimNadareishvili, IliaCernasev, AlinaNaik, Aanand DinkarChang, ChiachuNaim, SigalChen, Wei-MinNakagawa, KazuharuCheng, Winnie L. S.Nakayama, EnriChiarini, GiovanniNampoothiri, MadhavanChikara, Rupesh KumarNash, PaulChilicka-Hebel, KarolinaNebbioso, MarcellaChu, Nain-FengNho, Jae-HwiCicolin, AlessandroNiznik, JoshuaCiubara, Alexandru BogdanOkoye, ChukwumaClark, Heather M.Okpalauwaekwe, UdokaCorica, FrancescoOng, Adrian W.Costa, DavideOng, TerenceCowley, AlisonOoi, Theng ChoonCrispim, JoséOrange, Samuel T.D’Iorio, AlfonsinaPac, AgnieszkaDaniel, FernandaPage, AmyDariane, CharlesPalermo, SaraDasgupta, MonidipaPanfoli, IsabellaDavelaar, Eddy J.Pankowski, DanielDe Groot, EricPark, JunsooDe Melo, Rosa Cândida Carvalho PereiraParra-Rizo, María AntoniaDe Waal, Margot W. M.Pasipanodya, Elizabeth C.De, Pace VanessaPelotti, SusiDelellis, NailyaPepera, GaryfalliaDesai, PingalPérez-Ros, PilarDi Iorio, AngeloPérez-Zepeda, Mario UlisesDi Rosa, MirkoPetrashevich, InnaDoong, Jia-YaoPhyo, Aung ZawDubashynskaya, Natallia V.Phyo, Kyaw MyintEdelman, Linda S.Pikkel, JosephElío, IñakiPimentel, GustavoEllerkmann, RichardPodila, PradeepElvers, GregPodovšovnik, EvaEnrico, Gherlone FelicePolewczyk, AnnaErcoli, TommasoPowers, James S.Espinosa, EmmaPrasuhn, JannikEspoz-Lazo, SebastiánPrintza, AthanasiaEstgfaeller, NoraRababa, MohammadFemiano, FeliceRamalho, AndréFeola, AlessandroRapoport, MarkFernandes, Alan LinsRashid, UsmanFernandez, Bernadette O.Rodríguez-Hurtado, DianaFilbet, MarilèneRomano, MaurizioFirmino, HoratioRosi, AlessiaFrohnhofen, HelmutRossi, LorenaFuchs, Judith A.Roth, Klaus EdgarFullgrabe, ChristianRothenberg, ElisabetFulmer, TerrySagnelli, CaterinaFurtado, GuilhermeSánchez-González, DiegoGaggero, AlessioSánchez-Izquierdo, MacarenaGaines, Peter C.San-Segundo, RubénGallagher, DavidSantamaría-Peláez, MirianGallucci, AlessiaSantos-Rocha, Rita AlexandraGaspar, PhyllisSanz-Paris, AlejandroGay, AlainSarsak, HassanGendron, TraceyScarfone, Steven R.Georgiev, DejanScarlata, SimoneGermain, IsabelleSchreiner, NathanialGerymski, RafałScott, Judith M.Ghisletta, PaoloScott, Theresa L.Giménez-Llort, LydiaScutt, GregGiuliani, DanielaShelley, MackGómez-Delgado, GuillermoShimizu, YujiGonzález-Bernal, JerónimoShin, SunghoonGonzález-Moro, Ignacio MartínezShort, LeonieGonzalez-Santos, JosefaSikora, MaciejGoodman, ElizabethSimard, MarcGoroll, Allan H.Simmonds, BethanyGrandi, AnnalisaSingh, Desh DeepakGreiner, PhilipSingh, Devinder Kaur AjitGuenther, UlfSinvani, Liron DanayGüzel, RenginSobieszczanska, MalgorzataGuzsvinecz, TiborSolís García del Pozo, JuliánHaase, KristenSong, DanHakeem, Faisal F.Sotomayor, Camilo G.Hall, SallySousa, Ana S.Halvorsen, KjartanStein, GregorHan, SolaStrath, LarissaHansen, Troels K.Stümpel, JohanneHaragus, HoriaSuchánek, JakubHarvey, JoanSufaru, Irina-GeorgetaHenriques, Maria AdrianaSuksatan, WanichHeyn, PatriciaSulejczak, DorotaHodgson, PhilipSuñer, Soler Maria RosaHollanda, Ana DeSuslo, RobertHom, ChristySymmank, JuditHorgan, GrahamSzabo, Dan-AlexandruHorowitz, John D.Szluz, BeataHowell, BrittenyTanaka, GoroHsu, Chin-HsienTarantino, UmbertoHuijsman, RobbertTarashi, SamiraHuljenić, DarkoTarazona Santabalbina, Francisco JoséIida, TakatoshiTarozzi, AndreaIkuta, ToshikazuTarrant, SethIrazusta, JonTassone, BrunoIriuchishima, HironoTayek, John A.Ishizawa, YumikoTeixeira-Lemos, Editeİşsever, HalimTenhunen, MirjaJackson, Bianca N.Teramoto, ShinjiJauhiainen, MattiThalamuthu, AnbupalamKarachrysafi, SofiaThiamwong, LaddaKasselimis, DimitriosThieken, FranziskaKeilman, LindaThomas, Roger E.Kessler, ElizabethThompson, Lara A.Khan, Hafiz T. A.Tibor, HortobagyiKim, Hye-RyoungTiisanoja, AnttiKnobf, TishTorres, BlancaKoks, SulevToscano, MassimilianoKokubun, KeisukeTroutman-Jordan, MeredithKołodziej, MałgorzataTsartsalis, AthanasiosKostka, JoannaTurnbull, NiruwanKostka, TomaszTur-Sinai, AviadKotiya, DeepakUmemura, AkiraKozela, MagdalenaVaismoradi, MojtabaKrause, Jane E.Valdés-Badilla, PabloKujawski, SławomirValvani, RachnaKulnik, Stefan TinoVarju, CeciliaLai, Eddie Hsiang-HuaVecchia, Laura Adelaide DallaLane, HeatherVenturelli, MassimoLanger, FlorianVerduzco-Aguirre, Haydeé CristinaLarner, Andrew J.Vermeersch, PatriciaLeal, Ermelindo C.Vigezzi, Giacomo PietroLeBlanc, Raeann G.Villarejo, Mayor John JairoLeite, RichardVintila, MonaLeone, Paolo MariaVostrý, MichalLeonidis, AsteriosWalker, Robbie James EadesLesyk, LiliaWang, Tzu-ChuehLewko, JolantaWańkowicz, PawełLi, WeixinWard, KatherineLiampas, IoannisWaszyk-Nowaczyk, MagdalenaLietzen, Lone WintherWei, XinyuanLimpawattana, PanitaWijnen, VionaLin, Mei-ChenWilson, JasonLiotta, GiuseppeWittmann, MariaLippi, LorenzoWu, I-ChienLiu, JiXiong, JuyangLocatelli, Débora Regina SchneiderXu, HongyangLongobucco, YariYang, Nan-PingLópez-Gil, José FranciscoYang, YifanLorenz, Walter AugustYen, Chun-PoLotko, AleksanderYuichi, NishikawaLuchian, IonutZhu, BoWei

